# Cu(I)/Ionic Liquids Promote the Conversion of Carbon Dioxide into Oxazolidinones at Room Temperature

**DOI:** 10.3390/molecules24071241

**Published:** 2019-03-29

**Authors:** Jikuan Qiu, Yue Zhao, Yuling Zhao, Huiyong Wang, Zhiyong Li, Jianji Wang, Tiantian Jiao

**Affiliations:** 1 Collaborative Innovation Center of Henan Province for Green Manufacturing of Fine Chemicals, Key Laboratory of Green Chemical Media and Reactions, Ministry of Education, School of Chemistry and Chemical Engineering, Henan Normal University, Xinxiang 453007, Henan, China; qiujikuan001@163.com (J.Q.); zhaoyuechem@126.com (Y.Z.); whyhnxx@163.com (H.W.); lizhiyong03@126.com (Z.L.); 2 College of Chemical and Environmental Engineering, Shandong University of Science and Technology, Qingdao 266590, Shandong, China; jiaotiantian1988@163.com

**Keywords:** carbon dioxide, ionic liquids, catalysis, oxazolidinones

## Abstract

Recently, the efficient chemical fixation of carbon dioxide (CO_2_) into high value chemicals without using noble metal catalysts has become extremely appealing from the viewpoint of sustainable chemistry. In this work, a one-pot three component reaction of propargylic alcohols, anines and CO_2_ that can proceed in an atom economy and environmentally benign manner by combination of CuI and tetrabutylphosphonium imidazol ([P_4444_][Im]) as a catalyst was described. Catalysis studies indicate that this catalytic system is an effective catalyst for the conversion of CO_2_ into oxazolidinones at room temperature and ambient pressure without any solvent. The results provide a useful way to design novel noble metal-free catalyst systems for the transformation of CO_2_ into other valuable compounds.

## 1. Introduction

With the global consumption of fossil fuels, the increasing concentration of CO_2_ in the atmosphere could have a significant impact on the global climate [1,2]. Therefore, the development of efficient strategies to reduce CO_2_ emissions has becoms an imperative task for scientific researchers [3,4,5]. The chemical fixation and transformation of CO_2_ into valuable chemicals is an ideal pathway for reducing CO_2_ emissions and fully utilize this cheap C_1_ source [6,7,8,9,10]. Thus, considerable efforts have been devoted to the exploration of efficient strategy for chemical utilization of CO_2_ to produce high-value chemical commodities [11,12,13,14,15,16].

Oxazolidinones are a class of nitrogen-containing heterocyclic compounds which have been used as organic intermediates, chiral additives, antibacterial drugs and muscle relaxants, etc. [17,18,19,20]. The synthesis of oxazolidinones between propargyl alcohol, CO_2_ and an amine is an important and atom-economic reaction for chemical utilization of CO_2_ [12,21]. However, the biggest obstacle is the lack of an effective catalyst to facilitate its activation and conversion, since CO_2_ is a highly oxidized and thermodynamically stable molecule [22]. In recent years, various metal catalysts [23,24,25,26,27,28] and metal-free catalysts [29,30] have been verified to be efficient for the reaction. Nevertheless, most of the catalytic systems usually required high temperatures, high pressures, toxic organic solvents and noble metals. Therefore, a cheap, green and highly active catalyst is urgently needed in order to make the reaction go smoothly at room temperature and atmospheric pressure.

**Scheme 1:**
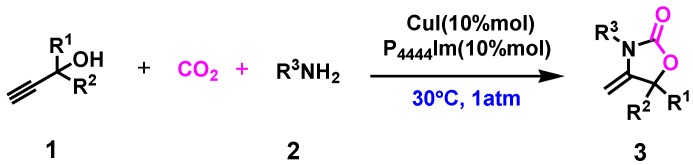
Cycloaddition reaction of propargyl alcohol and amines with CO_2_.

Our previous work has revealed that metal salts combined with ionic liquid (IL) represent a new hybrid catalyst that can be regarded as one of the most promising and efficient catalysts for chemical transformations of CO_2_ because of their remarkable synergistic catalysis mechanism [31]. For example, a Cu(I) salt/protic IL catalytic system can efficiently catalyze the cycloaddition of CO_2_ with propargylic alcohols to produce α-alkylidene cyclic carbonates under mild conditions. Inspired by these works, we now report that Cu(I) salt and the IL tetrabutylphosphonium imidazol([P_4444_][Im]) act as a catalyst for the three component reaction of terminal propargyl alcohols, CO_2_ and anines (Scheme 1). The results show that the CuI/[P_4444_][Im] catalyst system exhibits excellent catalytic performance for the reaction with a wide range of substrates under atmospheric pressure and room temperature conditions. In addition, the ionic liquid could be recovered and reused at least five times without an obvious loss of catalytic activity and selectivity.

## 2. Result and Discussion

**Figure 1:**
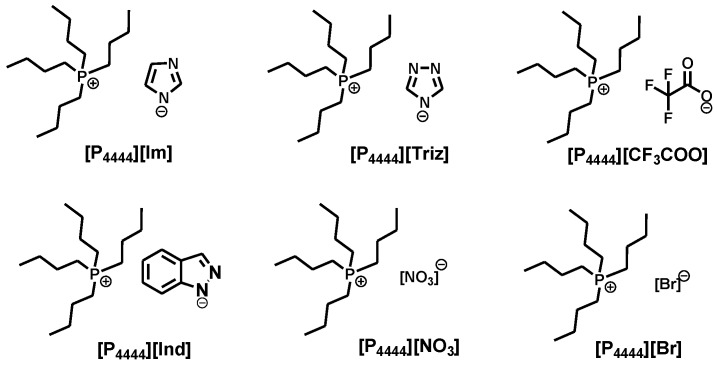
The structure and abbreviations of CO_2_-reactive ILs employed in this work.

Owing to the unique catalytic performance of ILs in CO_2_ conversion [32,33,34], several quaternary phosphonium-based ILs and various copper (I) salts were selected to catalyze the reaction. Figure 1 shows the structures of the ILs. 

The synthesis of 1,3-oxazolidin-2-one **3a** was carried out to systematically investigate the effect of various parameters on the reaction. The reaction was performed at 30 ^o^C, 1 atm CO_2_, and 24 h, and the results are shown in Table 1. As expected, the reaction did not occur in the absence of any catalysts. The individual IL [P_4444_][Im] and CuI as a catalyst were ineffective for the reaction, giving a low yield of product (Table 1, entries 2–3). To our delight, the yield of the product reached rose to 91% in the presence of CuI and [P_4444_][Im] (Table 1, entry 4). Encouraged by the result, we investigated the effect of the type of anion on the yield of **3a** in the reaction. The yield of 3a was found to be 70%, 41%, 35%, 30%, and 28%, respectively, when CuI/[P_4444_][Triz], CuI/[P_4444_][Ind], CuI/[P_4444_][CF_3_COO], CuI/[P_4444_][NO_3_] and [P_4444_][Br] were used as catalysts (Table 1, entries 5-9). The significant differences in their catalytic activity may be ascribed to the different nucleophilicity of these ILs as a result of their different basicity (Table S1). A larger IL pKa resulted in an improved absorption capacity of CO_2_ in the reaction system, which led to stronger reactivity between CO_2_ with propargylic alcohol, amine and the catalyst, to afford a higher product yield [35]. The result indicated that the ionic liquid anions play an important role in this conversion. Next, a variety of copper (I) salts with [P_4444_][Im] were used for this reaction. The activity of these copper salts followed the order: CuI > CuBr > CuCl (Table 1, entries 4, 10–11). Owing to the fact that among the halogenated metal salts the iodide anion had a stronger dissociation capacity, the metal cation and iodide anion could efficiently activate the substrates and promote the reaction in high yields [36]. The result suggested that the dissociation capacity of the anions acted a significant role in determining the catalytic activity of the halogenated copper salts. In addition, Cu_2_O and CuCN could also catalyze the reaction, affording moderate yields (65% and 32%, respectively) (Table 1, entries 12,13). Thus, CuI/[P_4444_][Im] was the best catalyst to further investigate this reaction.

**Figure 2:**
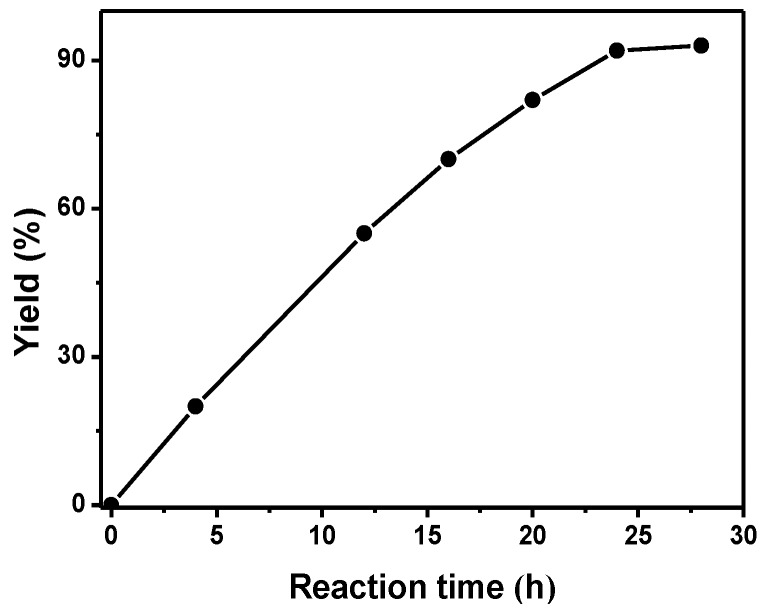
Effect of reaction time on the product yield over CuI and [P4444][Im]; Reaction conditions: **1a**, 1.0 mmol, **2a**, 1.0 mmol, CuI, 0. mmo, [P4444][Im], 0.1 mmol, CO_2_ (1 atm), 30 °C. The yield was isolated yield.

Subsequently, the influence of catalyst amount on the yield of product was studied at 30 ^o^C and 1 atm CO_2_ with a reaction time of 24 h. As seen in Figure S1, the yield of **3**a was strongly dependent on the catalyst amount, and a maximum yield of 90% was obtained in the presence of 10 mol% CuI. It was shown that the yield of **3a** will slowly decrease with further increase of the catalyst loading. Similar results were also obtained for the effect of IL amount in this reaction (Figure S2). Also, the dependence of reaction time on the yield of **3a** was studied and the results are shown in Figure 2. The reaction was carried out at 30 °C, 1atm CO_2_ using CuI (10 mol%) and [P_4444_][Im] (10mol%) as catalyst. It is shown that a yield of 92% could be obtained after 24h, and the yield of **3a** does not improve with further prolonged reaction time. Moreover, the effect of CO_2_ pressure on the reaction was studied. As shown in Table S2, the yield of **3a** increased significantly with increasing pressure. More CO_2_ could be dissolved in the liquid phase with further increasing pressure, which led to more contact between CO_2_ with the substrates and the catalyst, and thus a higher yield of product was obtained. From the above findings, the optimal reaction conditions for the reaction were: CuI/[P_4444_][Im] as the catalyst, 30 °C, 24 h, and 1 atm CO_2_.

The reactions of CO_2_ with different kinds of propargylic alcohols and amines were evaluated under the optimized reaction conditions, and the results are listed in Table 2. It was shown that the coupling reaction of different alkyl substituted propargylic alcohols with CO_2_ and anines proceeded smoothly, producing the corresponding oxazolidinones in good yields under mild conditions. It was noted that a moderate yield of product was obtained for the propargylic alcohol with a cyclohexyl group (**3i**), indicating the steric hindrance of the substituent seemed to hamper the reaction. Furthermore, we also investigate the effect of amines with various substituents in this transformation. It was clearly shown that most of alkyl-substituted amines were efficient substrates to produce the corresponding oxazolidinones. However, when the R^3^ group of amines was a phenyl group, no product was obtained (compound **3k**), even after a prolonged reaction time of 36 h. This phenomenon can be attributed to both the weak N-nucleophilicity and steric hindrance of the substrates, which further hamper the reaction [37]. From the above analysis, the catalyst system reported here is more favorable for alkyl amines than aromatic amines.

**Figure 3:**
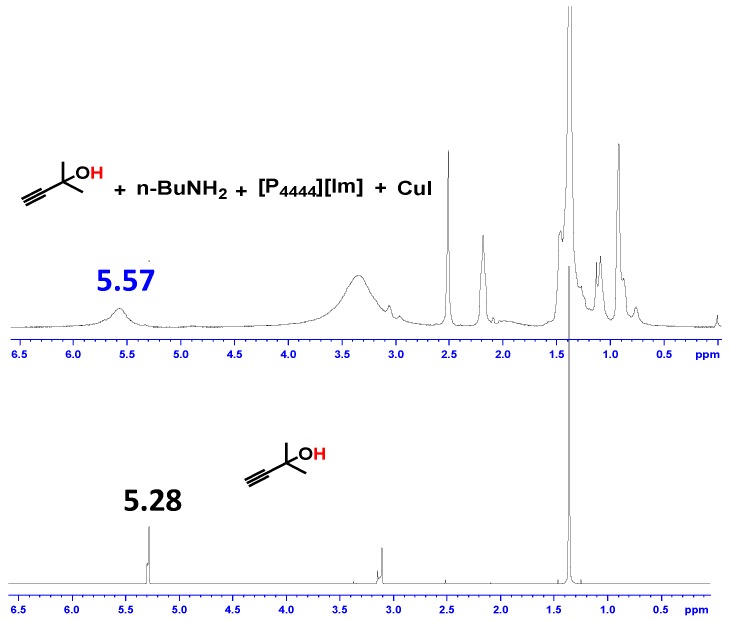
^1^H-NMR spectra of propargylic alcohol, *n*-BuNH_2_ with and without CuI/[P_4444_][Im] catalyst.

**Figure 4:**
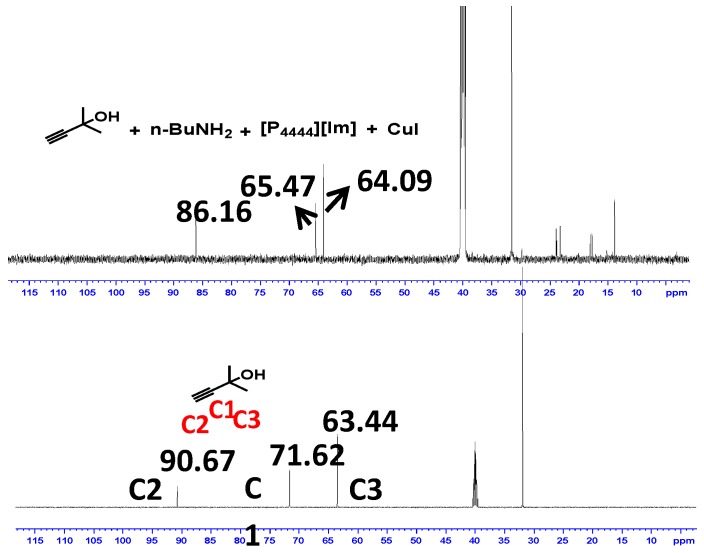
^13^C-NMR spectra of propargylic alcohol, *n*-BuNH_2_ with and without CuI/[P_4444_][Im] catalyst.

**Scheme 2:**
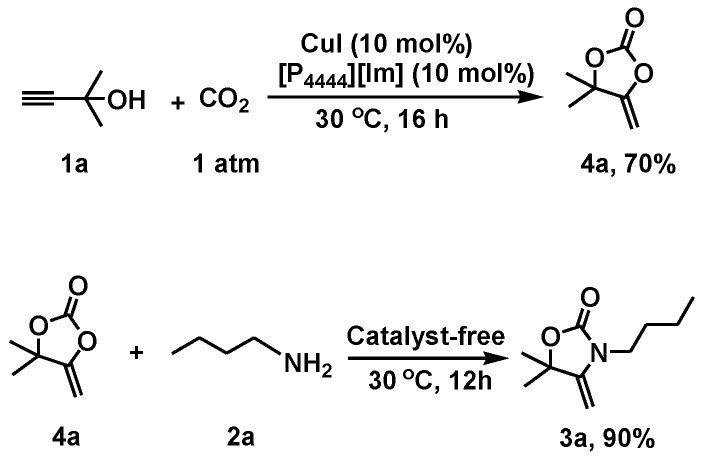
Control experiments.

To gain insight into the reaction mechanism, the catalytic role of [P_4444_][Im]/CuI with CO_2_ and the substrates were elucidated by ^1^H-NMR and ^13^C-NMR spectroscopy. In the ^1^H-NMR spectrum of the mixture of [P_4444_][Im]/CuI with **1a** and *n*-BuNH_2_ (Figure 3), the OH signal became broad and shifted from 5.28 to 5.57, which indicated the formation of a hydrogen bond between [P_4444_][Im] and **1a [35]**. In addition, the ^13^C-NMR signal assigned to the C1, C2, and C3 sites could be attributed to the interaction between CuI with the C≡C bond, leading to the activation of propargylic alcohol (Figure 4). These analysis suggested that the -OH and C≡C bond on the substrate **1a** were synergistically activated by [P_4444_][Im] and CuI. Moreover, several additional control experiments were performed and the results are shown in Scheme 2. It was shown that the CuI/[P_4444_][Im] catalyst could efficiently catalyze the reaction of **1a** with CO_2_ to form cyclic carbonate **4a** with a yield of 70%. In addition, **4a** reacted with *n*-butylamine to afford **3a** smoothly without any catalyst. Therefore, the reaction of propargylic alcohols, anines and CO_2_ can be assumed to go through the cyclic carbonate pathway [37].

**Scheme 3:**
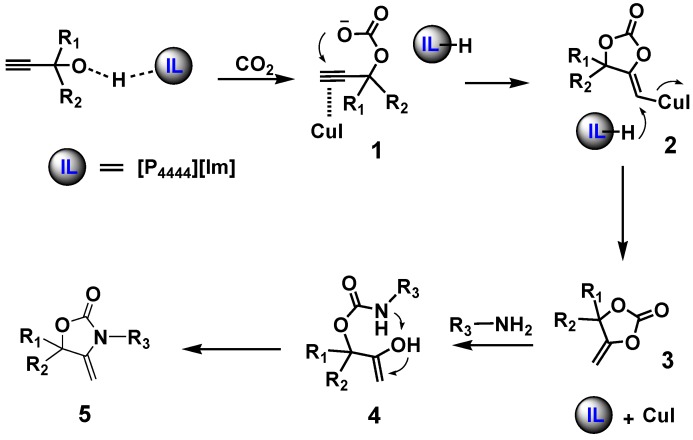
The proposed mechanism for the reaction of propargyl alcohol, CO_2_ and an amine catalyzed by [CuI]/[P_4444_][Im].

Based on the above results, a plausible mechanism for the reaction catalyzed by CuI/[P_4444_][Im] as depicted in Scheme 3 is proposed. Firstly, the hydroxyl group of the propargyl alcohol is activated by IL [P_4444_][Im], then it reacts with CO_2_ to generate a zwitterionic carbamate species **1**. Meanwhile, the C≡C triple bond of the propargyl alcohol was activated by CuI, and then the intermediate **2** was obtained. Then the intermediate cyclic carbonate **3** was formed by an intramolecular ring-closing reaction with the release of the catalyst. Finally, the cyclic carbonate undergoes a nucleophilic attack by a primary amine to generate the product oxazolidinone **5**.

## 3. Experimental Section

### 3.1. Chemicals

Propargylic alcohols, amines, CuI, Cu_2_O, CuCl, CuBr, CuCN were obtained from J&K Scientific Company Limited (Beijing, China). The ILs [P_4444_][CF_3_COO], [P_4444_][NO_3_] and [P_4444_][Br] were purchased from Lanzhou Greenchem ILs (Lanzhou, Gansu, China). Other ILs such as [P_4444_][Triz], [P_4444_][Ind] and [P_4444_][Im] were synthesized by the ion exchange method [38].

### 3.2. General Procedures for the Synthesis of Oxazolidinones

Typically, propargylic alcohol **1a** (1.0 mmol), *n*-butylamine (**2a**, 1.0 mmol) and CuI (0.1 mmol)/[P_4444_][Im] (0.1 mmol) were mixed in a 10 mL Schlenk flask. Then the reaction mixture was stirred at 30 °C for 24 h in the pressure of 0.1 MPa CO_2_. After the reaction was completed, water (5 mL) was added into this mixture, and the organic phase was purified through column chromatography. The ionic liquid was collected and reused without further treatment by removing any water under vacuum.

### 3.3. Characterization of the Products

The products were confirmed by using ^1^H-NMR analysis on Avance NEO 400 spectrometer (Bruker, Beijing, China) and Avance III HD 600 spectrometer (Bruker, Beijing, China). All of the products matched well with the previously reported experimental results [26].

*3-butyl-5,5-dimethyl-4-methyleneoxazolidin-2-one* (**3a**), orange oil; ^1^H-NMR (CDCl_3_, 400 MHz does not match above) *δ*: 4.07 (d, *J* = 4.0 Hz, 1 H), 3.98 (d, *J* = 4.0 Hz, 1 H), 3.44 (t, *J* = 8.0 Hz, 2 H), 1.61–1.52 (m, 2 H), 1.49 (s, 6 H), 1.37–1.29 (m, 2 H), 0.95 (t, *J* = 7.2 Hz, 3 H) ppm.

*3-butyl-5-isobutyl-5-methyl-4-methyleneoxazolidin-2-one* (**3b**), orange solid; ^1^H-NMR (CDCl_3_, 600 MHz) *δ*: 4.09 (s, 1 H), 3.93 (s, 1 H), 3.51-3.38 (m, 2 H), 1.80–1.43 (m, 8 H), 1.40–1.31 (m, 2 H), 1.02–0.89 (m, 9 H) ppm.

*3-cyclohexyl-5,5-dimethyl-4-methyleneoxazolidin-2-one* (**3c**), orange solid; ^1^H-NMR (CDCl_3_, 600 MHz) *δ*: 4.19 (d, *J* = 3.0 Hz, 1 H), 3.97 (d, *J* = 3.0 Hz, 1 H), 3.55 (t, *J* = 12.0 Hz, 1 H), 1.86-1.66 (m, 13 H), 1.25–1.14 (m, 4 H) ppm.

*3-isobutyl-5,5-dimethyl-4-methyleneoxazolidin-2-one* (**3d**), colorless oil; ^1^H-NMR (CDCl_3_, 600 MHz) δ: 4.27 (s, 1 H), 3.11–3.04 (m, 1 H), 2.96–2.89 (m, 1 H), 1.99-1.92 (m, 1 H), 1.51 (s, 3 H), 1.43. (s, 2 H), 1.34 (d, *J* = 12 Hz, 2 H), 0.92–0.87 (m, 6 H) ppm.

*3-hexyl-5,5-dimethyl-4-methyleneoxazolidin-2-one* (**3e**), colorless oil; ^1^H-NMR (CDCl_3_, 600 MHz) δ: 4.07 (d, *J* = 2.4 Hz, 1 H), 3.97 (d, *J* =3 Hz, 1 H), 3.43 (t, *J* = 7.8 Hz, 2 H), 1.63–1.58 (m, 2 H), 1.49 (s, 1 H), 1.32–1.30 (m, 6H), 0.89–0.88 (m, 3H) ppm.

*5-ethyl-3-isobutyl-5-methyl-4-methyleneoxazolidin-2-one* (**3f**), colorless oil; ^1^H-NMR (CDCl_3_, 600 MHz) δ: 4.10 (d, *J* = 3 Hz, 1 H), 3.92 (d, J = 3 Hz, 1 H), 3.30–3.19 (m, 2 H), 1.85–1.80 (m, 1 H), 1.48–1.45 (d, *J* = 7.2 Hz, 5 H), 1.02 (t, *J* = 7.8 Hz, 2 H), 0.93–0.90 (m, 6 H), 0.89 (s, 1 H)ppm.

*3,5-diisobutyl-5-methyl-4-methyleneoxazolidin-2-one* (**3g**), orange solid; ^1^H-NMR (CDCl_3_, 600 MHz) *δ*: 4.09 (s, 1 H), 3.93 (s, 1 H), 3.28–3.23 (m, 2 H), 2.14–2.04 (m, 1 H), 1.51 (s, 1 H), 1.04–0.98 (m, 2 H), 0.97–0.91 (m, 12 H) ppm.

*3-cyclohexyl-5-isobutyl-5-methyl-4-methyleneoxazolidin-2-one* (**3h**), orange solid; ^1^H-NMR (CDCl_3_, 600 MHz) *δ*: 4.24 (s, 1 H), 3.93 (s, 1 H), 3.64–3.53 (m, 1 H), 2.15–1.45 (m, 12 H), 1.34–1.15 (m, 4 H), 0.94–0.91 (m, 6 H) ppm.

*3-cyclohexyl-5-ethyl-5-methyl-4-methyleneoxazolidin-2-one* (**3i**), orange solid; ^1^H-NMR (CDCl_3_, 600 MHz) *δ*: 4.25 (d, *J* = 3.0 Hz, 1 H), 3.93 (d, *J* = 3.0 Hz, 1 H), 3.62–3.55 (m, 1 H), 1.66–1.47 (m, 11 H), 1.35–1.18 (m, 4 H), 0.88 (t, *J* = 7.8 Hz, 3 H) ppm.

*3-Butyl-4-methylene-1-oxa-3-azaspiro[4.5]decan-2-one* (**3j**), orange oil; ^1^H-NMR (CDCl_3_, 600 MHz) *δ*: 4.07 (d, *J* = 3.0 Hz, 1 H), 3.95 (d, *J* = 2.4 Hz, 1 H), 3.43 (t, *J* = 7.8 Hz, 2 H), 1.76–1.65 (m, 5 H), 1.61–1.50 (m, 7 H), 1.40–1.31 (m, 2 H), 0.94 (t, *J* = 7.2 Hz, 3 H) ppm.

## 4. Conclusions

In conclusion, we have developed an excellent and cost-competitive catalytic process catalyzed by the CuI/[P_4444_][Im] system for the transformation of CO_2_ to form oxazolidinones. The reaction can proceed efficiently under room temperature and atmospheric pressure conditions to give high yields of product. A wide range of propargylic alcohols and amines has been employed, which confirmed the versatility of the catalyst system for the synthesis of oxazolidinones. Preliminary mechanistic studies suggest that the reaction of propargylic alcohols, anines and CO_2_ could involve the cyclic carbonate pathway. This work reveals the great potential of ionic liquids combined with metal salts as an efficient type of catalysts for CO_2_ conversion under mild conditions.

## Figures and Tables

**Table 1:** Reaction of 2-methyl-3-butyn-2-ol (**1a**), *n*-butylamine (**2a**) and CO_2_ in various catalyst systems ^a^.

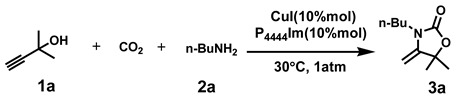			
Entry	Catalyst	IL	Yield/% ^b^
1	_^c^	_^d^	0
2	_^c^	[P_4444_][Im]	5
3	CuI	_^d^	34
4	CuI	[P_4444_][Im]	91
5	CuI	[P_4444_][Triz]	70
6	CuI	[P_4444_][Ind]	41
7	CuI	[P_4444_][CF_3_COO]	35
8	CuI	[P_4444_]NO_3_	30
9	CuI	[P_4444_]Br	28
10	CuBr	[P_4444_][Im]	71
11	CuCl	[P_4444_][Im]	42
12	Cu_2_O	[P_4444_][Im]	65
13	CuCN	[P_4444_][Im]	32
Entry	Catalyst	IL	Yield/% ^b^

^a^ Reaction condition: **1a**, 1.0 mmol, **2a**, 1.0 mmol, catalyst, 0.1 mmol, CO_2_ (0.1 MPa, 99.999%), 24 h, 30 °C; ^b^ Isolated yieid; ^c^ Without copper salt; ^d^ Without IL.

**Table 2:** Three-component reactions of propargylic alcohols (**1**), amines (**2**) and CO_2_ under optimized conditions ^a^.

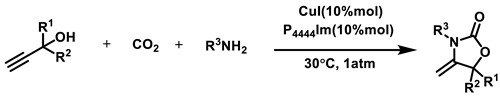			
**1**	**2**	**3**	
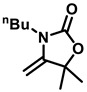	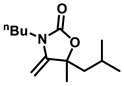	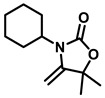	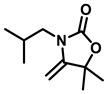
**3a (87%) ^b^**	**3b (81%) ^b^**	**3c (82%) ^b^**	**3d (85%) ^b^**
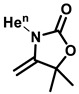	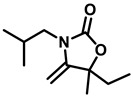	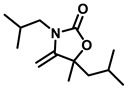	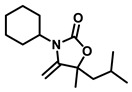
**3e (81%) ^b^**	**3f (70%) ^b^**	**3g (75%) ^b^**	**3h (73%) ^b^**
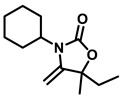	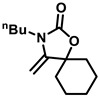	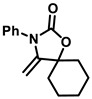	
**3i (71%) ^b^**	**3j (54%) ^b^**	**3k (0) ^c^**	

^a^ Reaction conditions: **1**, 1.0 mmol, **2**, 1.0 mmol, CuI, 0.1 mmol, [P_4444_][Im], 0.1 mmol, CO_2_ (1 atm), within 24 h, 30 °C. ^b^ Isolated yieid. ^c^ The reaction time is prolonged to 36 h.

## Supplementary Materials

The following are available online.

## Abbreviations

[P_4444_][Im]	Tetrabutylphosphonium imidazole
[P_4444_][CF_3_COO]	Tetrabutylphosphonium 2,2,2-trifluoroacetic acid
[P_4444_][Triz]	Tetrabutylphosphonium 1,2,4-triazole
[P_4444_][Ind]	Tetrabutylphosphonium indazole
[P_4444_][Br]	Tetrabutylphosphonium bromide
[P_4444_][NO_3_]	Tetrabutylphosphonium nitrate
