# Supplementary material for: Cu(I)/Ionic Liquids Promote the Conversion of Carbon Dioxide into Oxazolidinones at Room Temperature
Source: Molecules. 2019 Mar 29;24(7):1241. doi: 10.3390/molecules24071241 (PMC6480545; doi:10.3390/molecules24071241)
Supplement: Supplementary file 1 [file molecules-24-01241-s001.pdf]

Supporting Information

Cu(I)/Ionic liquids Promoted the Conversion of Carbon Dioxide into Oxazolidinones at Room Temperature

Jikuan Qiu[1], Yue Zhao[1], Yuling Zhao[1,], Huiyong Wang[1], Zhiyong Li[1], Jianji Wang[1,*] and Tiantian Jiao[2]*

1, Collaborative Innovation Center of Henan Province for Green Manufacturing of Fine Chemicals, Key Laboratory of Green Chemical Media and Reactions, Ministry of Education, School of Chemistry and Chemical Engineering, Henan Normal University, Xinxiang, Henan 453007 (P. R. China)

E-mail: jwang@htu.cn; ylzhao@htu.cn

2, College of Chemical and Environmental Engineering, Shandong University of Science and Technology, Qingdao 266590, Shandong, China

Table of Contents

Section 1. Synthetic Procedures of the ILs 2

Section 2. Figures and Tables 3

Section 3. References 4

Section 4. NMR Spectra 6

Section 1: Synthetic Procedures of the ILs

[P_{4444}][Im], [P_{4444}][Triz] and [P_{4444}][Ind] were synthesized and purified by the following procedures[1-2]. As an example, the procedure for the preparation of [P_{4444}][Im] was described. First, [P_{4444}]OH (tetrabutylphosphonium hydroxide) was obtained from the raw material [P_{4444}]Br through the anion-cation exchange resin (water : ethanol=1:1). Equimolar Im was added, and the mixture was then stirred for 12 h at room temperature. After that, the solvent was distilled off at 45 ℃ by rotary evaporation. The obtained products were dried for at least 24 h under reduced pressure. The chemical structures of these ILs were confirmed by ^{1}H NMR and ^{13}C NMR spectroscopy. The data of products is consistent with the previously reported experimental results [3].

[P_{4444}][Im]. orange oil; ^{1}H NMR ($CDCl_3$, 400 MHz) δ: 7.56 (s, 1 H), 6.95 (s, 2 H), 2.30-2.14 (m, 8 H), 1.51-1.34 (m, 16 H), 0.88 (t, J = 8.0 Hz, 12 H) ppm; ^{13}C NMR ($CDCl_3$, 150 MHz) δ: 135.04, 23.68, 18.79, 18.48, 13.02 ppm.

[P_{4444}][Triz]. orange oil; ^{1}H NMR (DMSO-d_6, 400 MHz) δ: 17.67 (s, 2H), 2.22−2.12 (m, 8H), 1.51−1.34 (m, 16H), 0.92 (t, J=7.2 Hz, 12H) ppm; ^{13}C NMR ($CDCl_3$, 150 MHz) δ: 146.63, 145.89, 23.72, 23.62, 23.24, 18.50, 18.19, 13.17 ppm.

[P_{4444}][Ind]. orange oil; ^{1}H NMR ($CDCl_3$, 400 MHz) δ: 7.89 (s, 1H), 7.57 (d, J=8.0 Hz, 1H),7.06−6.75 (m, 3H), 2.22−2.11 (m, 8H), 1.52−1.34 (m, 16H), 0.92 (t, J= 6.8 Hz, 12H) ppm; ^{13}C NMR ($CDCl_3$, 150 MHz) δ: 158.86, 140.33, 133.76, 126.08, 123.06, 120.37, 110.58, 23.89, 23.54, 18.88, 18.58, 13.40 ppm.

Section 2: Figures

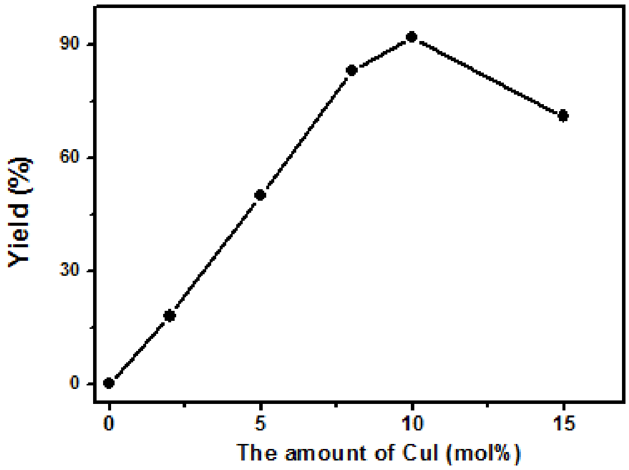

Figure S1. Influence of CuI amount on the yield of oxazolidinones. Reaction conditions: 1a (1 mmol), 2a (1mmol), [P_{4444}][Im] (10 mol%), CO_2 (1atm), 30 ˚C, 24 h. The yields were determined by ^{1}H NMR spectroscopy.

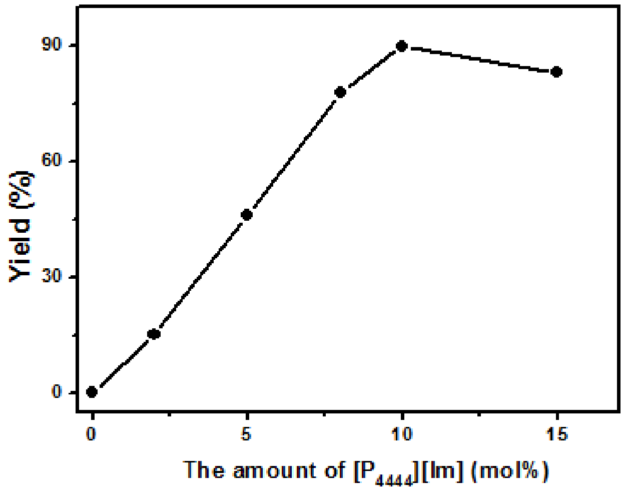

Figure S2. Influence of [P_{4444}][Im] amount on the yield of oxazolidinones. Reaction conditions: 1a (1 mmol), 2a (1mmol), CuI (10 mol%), CO_2 (1atm), 30 ˚C, 24 h. The yields were determined by ^{1}H NMR spectroscopy.

Table S1. The effect pKa of different ionic liquids on the synthesis of 3-butyl-5,5-dimethyl-4-methyleneoxazolidin-2-one (3a) from atmospheric CO_2 [a]

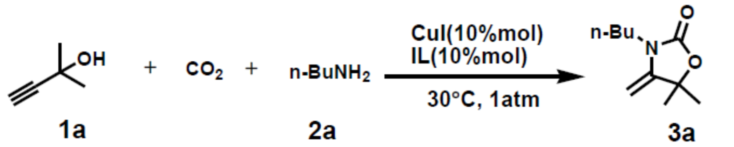

Entry	IL	pKa of IL in DMS0[4]	Yield/ % [b]
4	[P4444][Im]	18.6	91
5	[P4444][Triz]	13.9	70
6	[P4444][Ind]	--	41
7	[P4444][CF3COO	3.45	35
8	P4444]NO3	0.9	30
9	[P4444]Br	--	28

[a] Reaction condition: 2-methylbut-3-yn-2-ol (1a, 1.2 mmol), n-Butylamine (2a, 1.2mmol), CuI (0.10 mol), IL (0.10 mol),CO2 (0.1 MPa, 99.999%), 24 h, 30 °C; b The isolated yieid.

Table S2. Effect of CO2 pressure on the isolated yield of 3a.

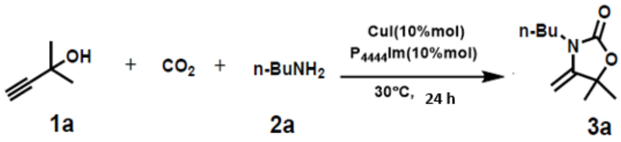

Entry	CO2 pressure	Yield (%) [b]
1	0	0
2	0.3	33
3	0.5	52
4	0.7	63
5	1.0	90

Reaction conditions: 1a (1 mmol), 2a (1 mmol), CuI (0.1 mmol), [P4444][Im] (0.1 mmol), 30 ºC, 24 h

Section 3: References

[1] Pei, X.; Xiong, D.; Pei, Y.; Wang, H.; Wang, J. Switchable oil–water phase separation of ionic liquid-based microemulsions by CO_2. *Green Chem*. **2018**, *20*, 4236-4244.

[2] Shi, Y.; Xiong, D.; Wang, H.; Zhao, Y.; Wang, J. Reversible switching of amphiphilic self- assemblies of ionic liquids between micelle and vesicle by CO_2. *Langmuir*, **2016**, *32*, 6895-6901.

[3] Zhao, Y.; Yang, Z.; Yu, B.; Zhang, H.; Xu, H.; Hao, L.; Han, B.; Liu, Z. Task specific ionic liquid and CO_2-cocatalysed efficient hydration of propargylic alcohols to a-hydroxy ketones. *Chem. Sci.* **2015**, *6*, 2297-2301.

[4] Bordwell, F. G. Equilibrium acidities in dimethyl sulfoxide solution. *Acc. Chem. Res.* **1988**, *21*, 456-463.

Section 4: NMR Spectra

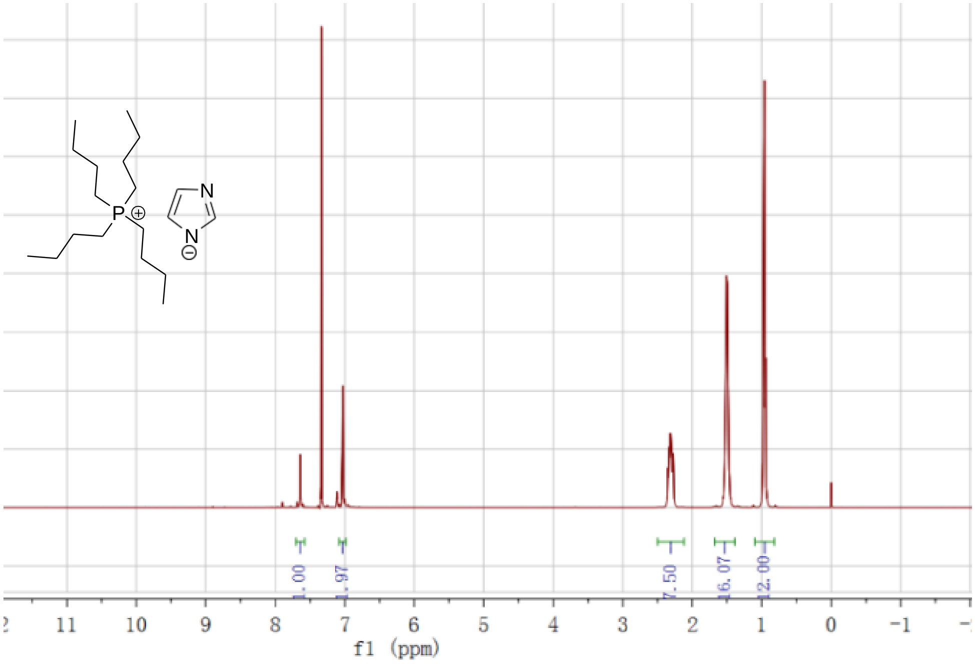

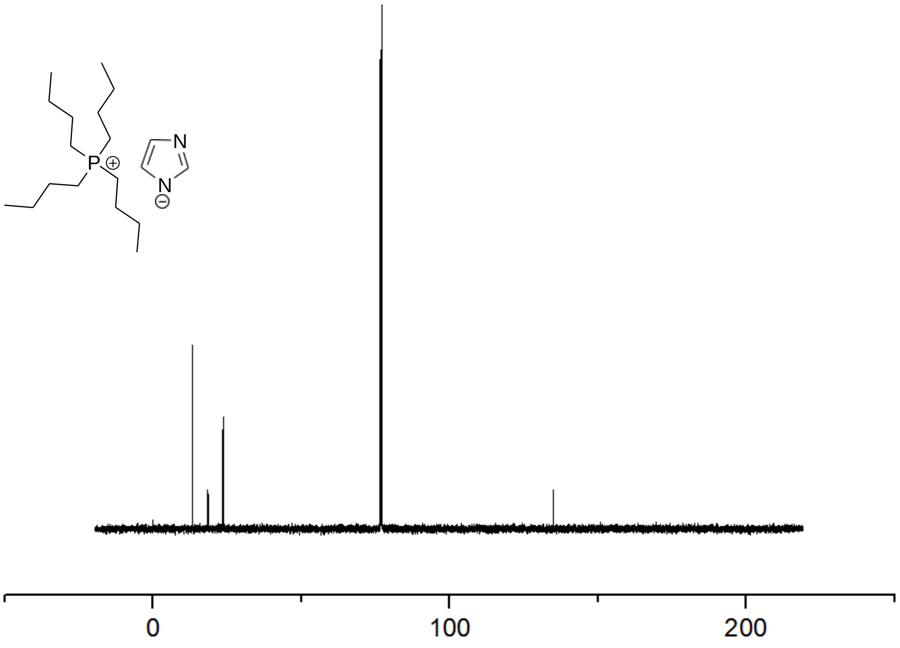

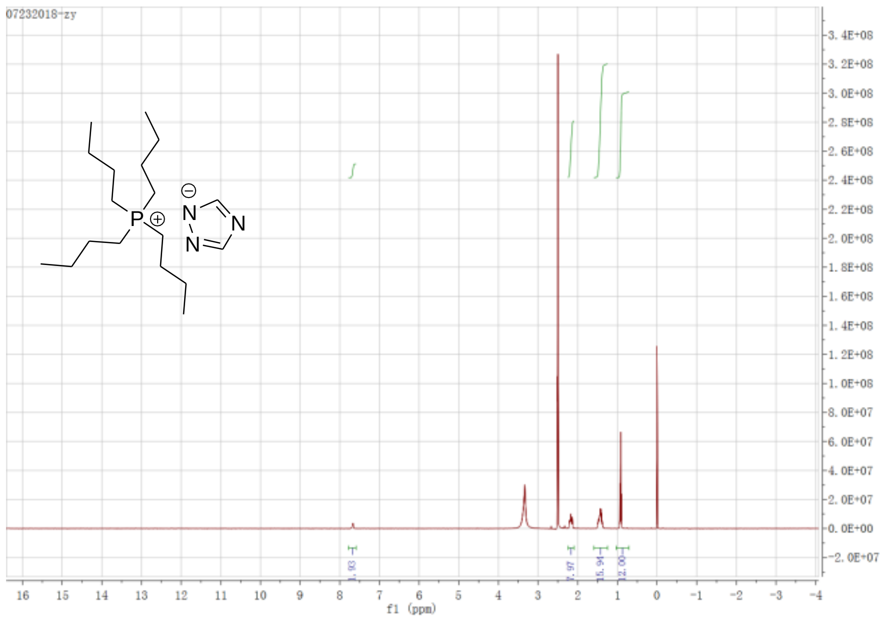

07232018-zy
P⊕
N
N
N
⊖
1.93
7.97
15.94
12.00
f1 (ppm)
16 15 14 13 12 11 10 9 8 7 6 5 4 3 2 1 0 -1 -2 -3 -4
3.4E+08
3.2E+08
3.0E+08
2.8E+08
2.6E+08
2.4E+08
2.2E+08
2.0E+08
1.8E+08
1.6E+08
1.4E+08
1.2E+08
1.0E+08
8.0E+07
6.0E+07
4.0E+07
2.0E+07
0.0E+00
-2.0E+07

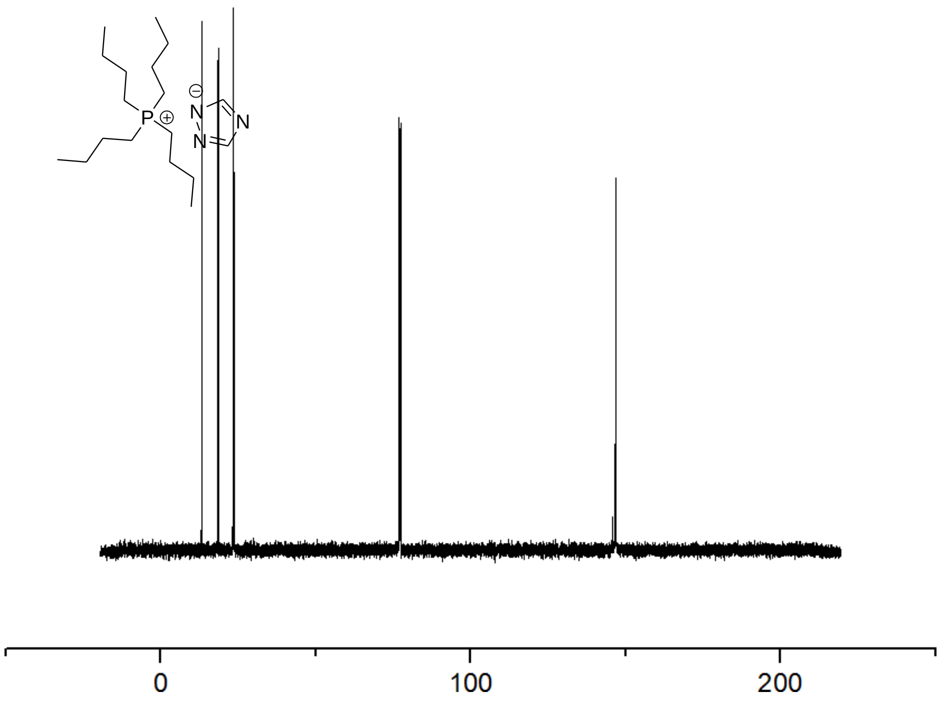

P⊕
N
N
N
⊖
0
100
200

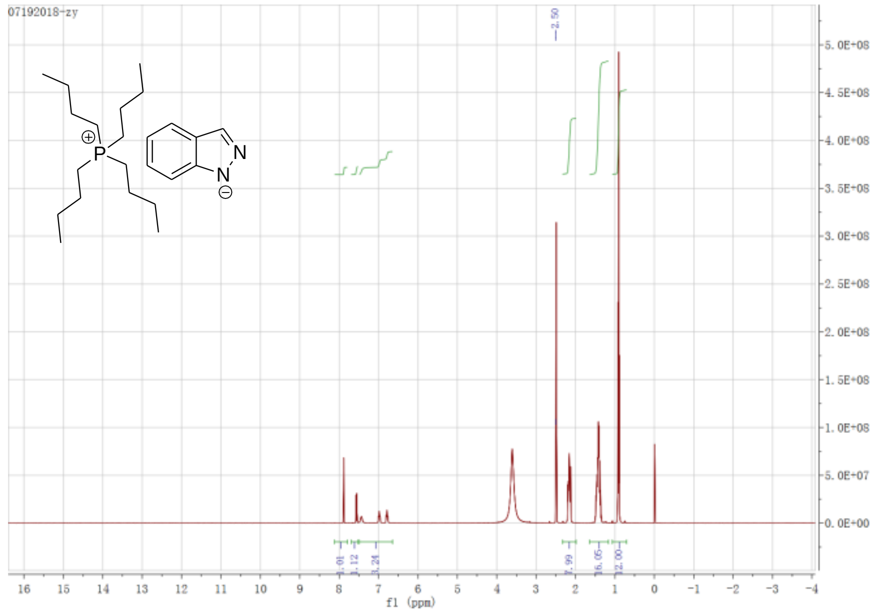
07192018-zy
2.50
P
N
N
1.01
1.12
3.24
7.99
16.05
12.00
f1 (ppm)

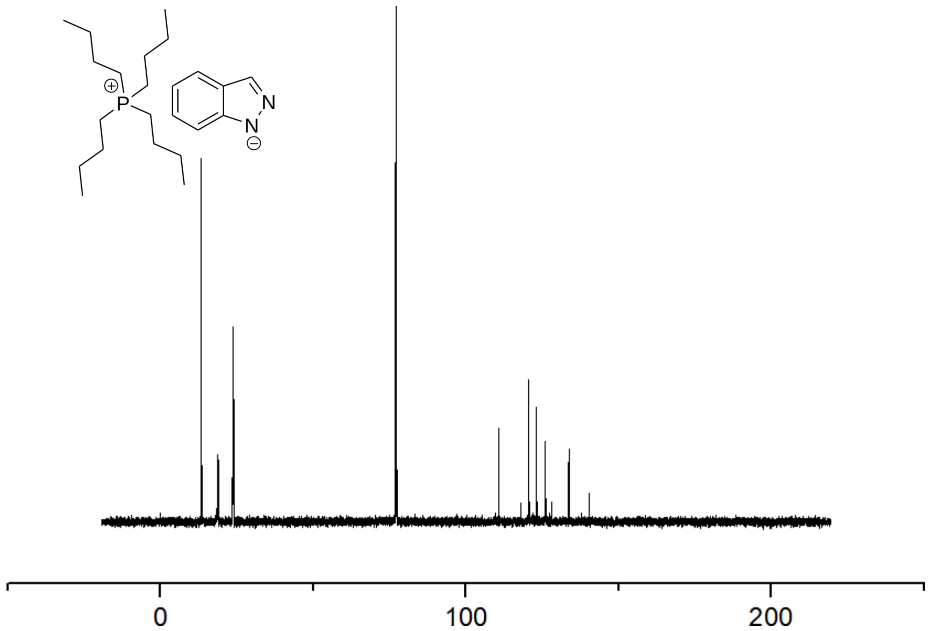
P
N
N
0
100
200

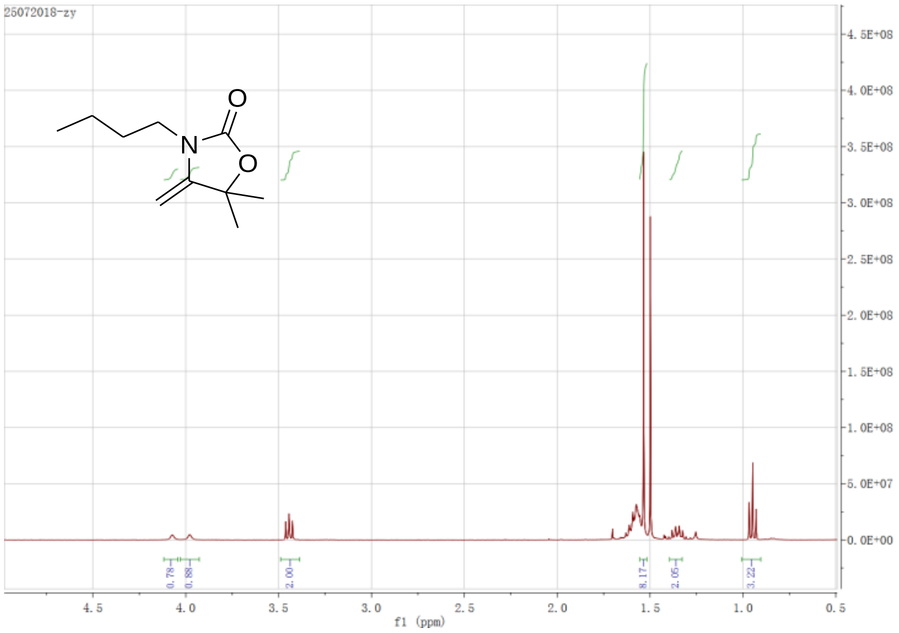

25072018-zy
O
N
O
0.78
0.88
2.00
8.17
2.05
3.22
4.5
4.0
3.5
3.0
2.5
2.0
1.5
1.0
0.5
f1 (ppm)
4.5E+08
4.0E+08
3.5E+08
3.0E+08
2.5E+08
2.0E+08
1.5E+08
1.0E+08
5.0E+07
0.0E+00

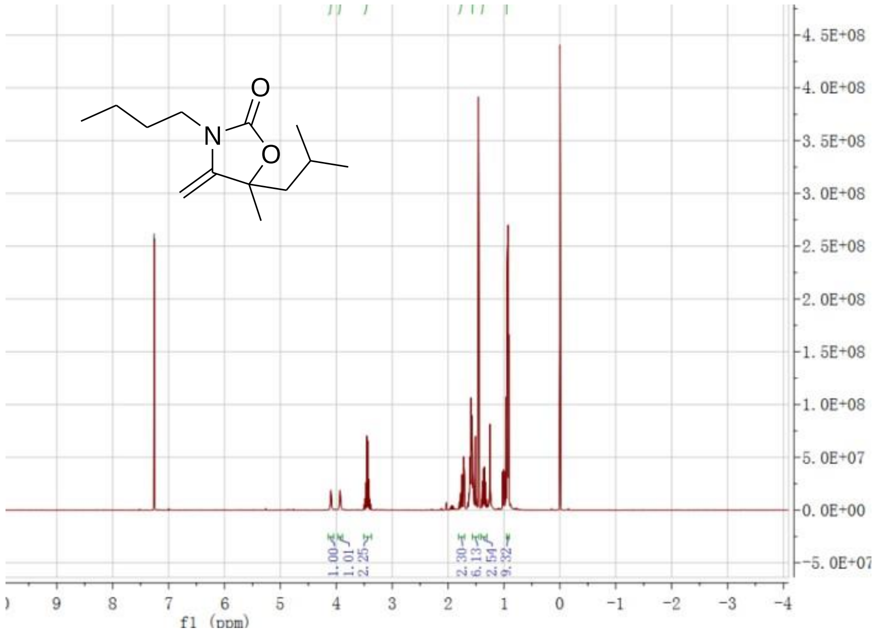

O
N
O
1.00
1.01
2.25
2.30
6.13
2.54
9.32
9
8
7
6
5
4
3
2
1
0
-1
-2
-3
-4
f1 (ppm)
4.5E+08
4.0E+08
3.5E+08
3.0E+08
2.5E+08
2.0E+08
1.5E+08
1.0E+08
5.0E+07
0.0E+00
-5.0E+07

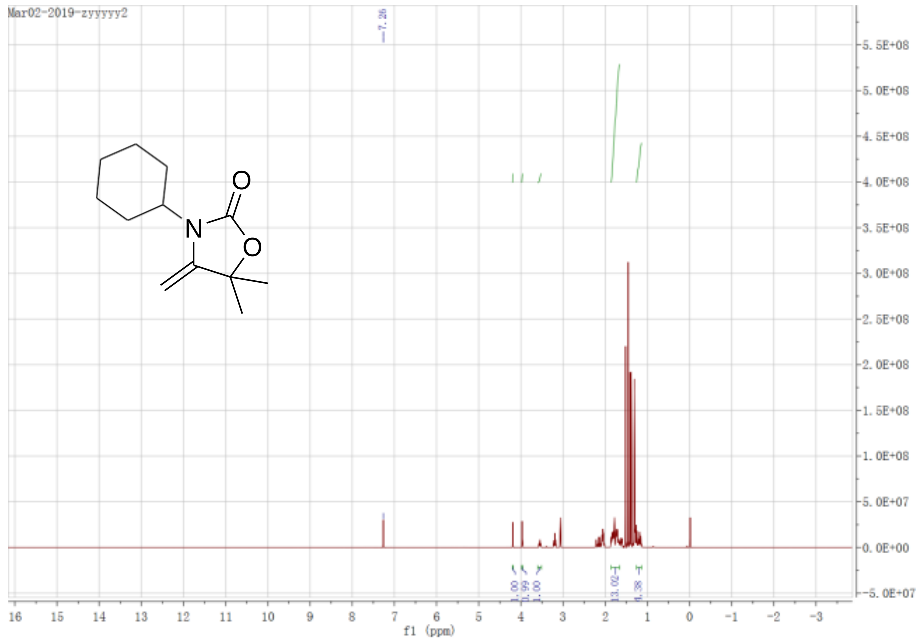
Mar02-2019-zyyyyy2
7.26
O
N
O
1.00
0.99
1.00
13.02
6.38
f1 (ppm)

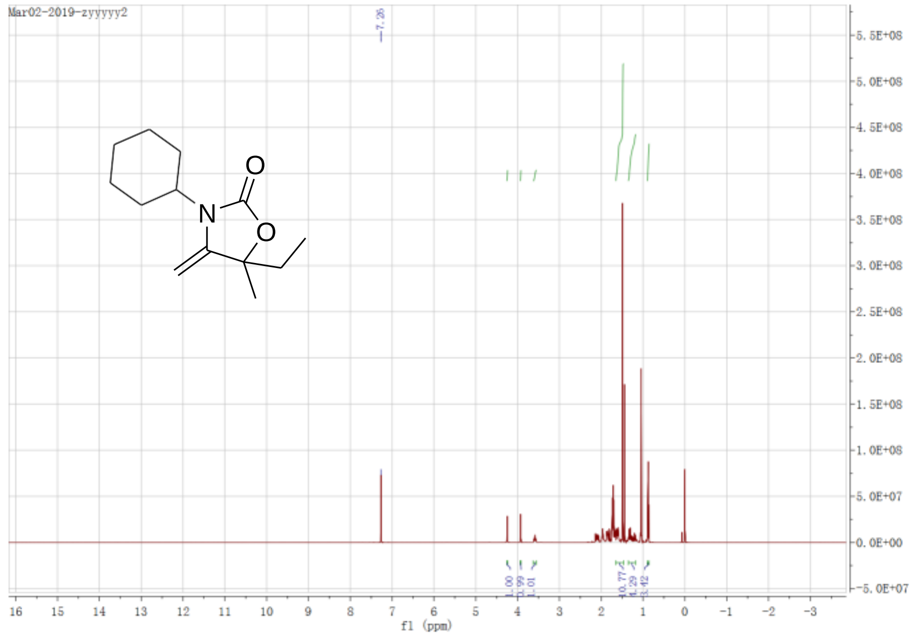
Mar02-2019-zyyyyy2
7.26
O
N
O
1.00
0.99
1.01
10.77
f1 (ppm)

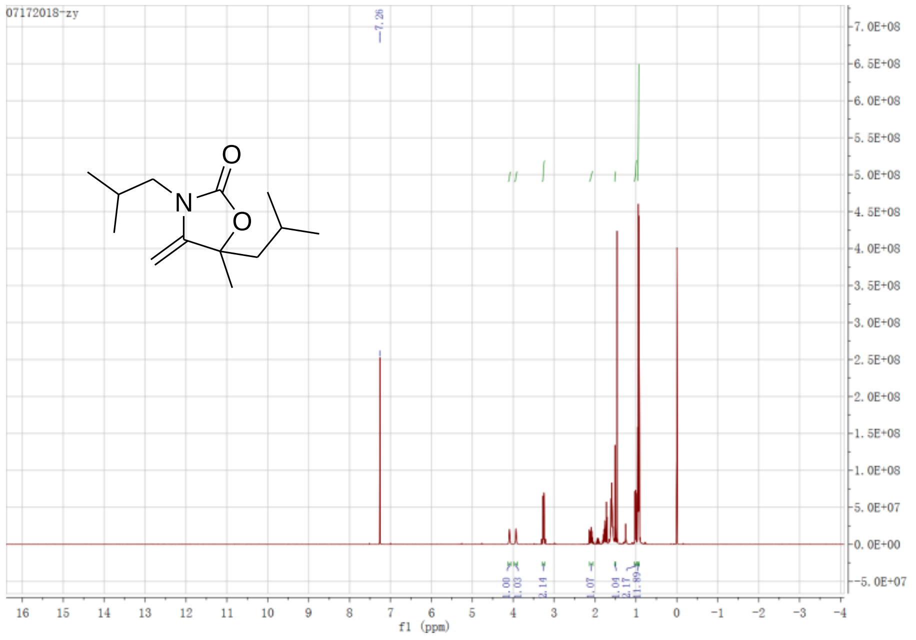
07172018-zy
7.26
1.00
1.03
2.14
1.07
1.04
2.17
11.89
f1 (ppm)
O
N
O

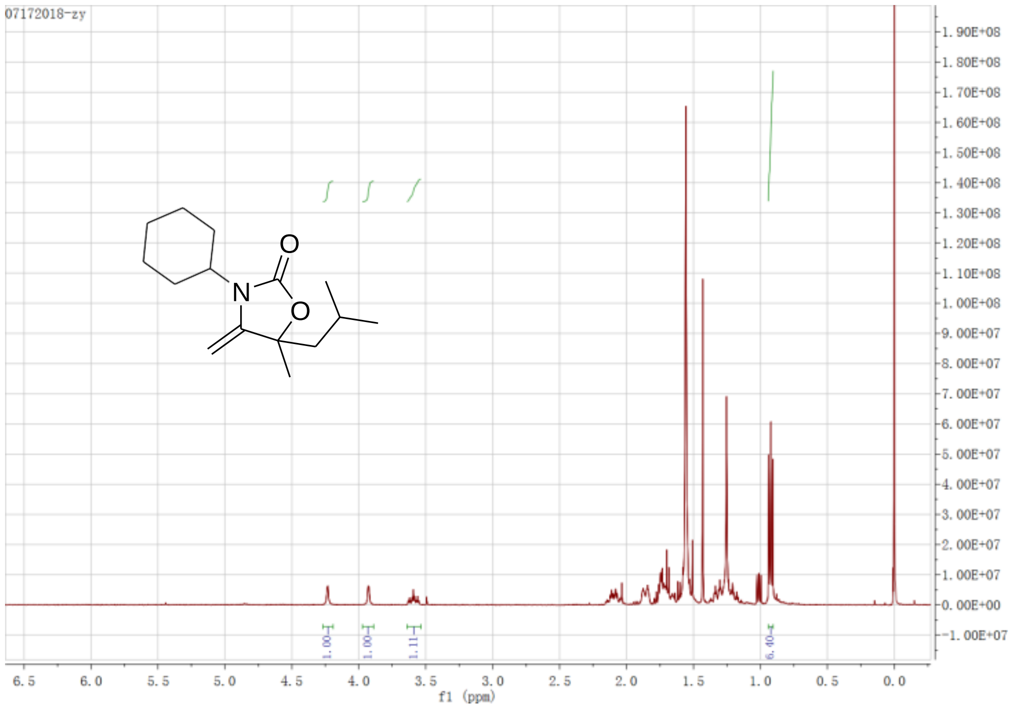
07172018-zy
1.00
1.00
1.11
6.40
f1 (ppm)
O
N
O

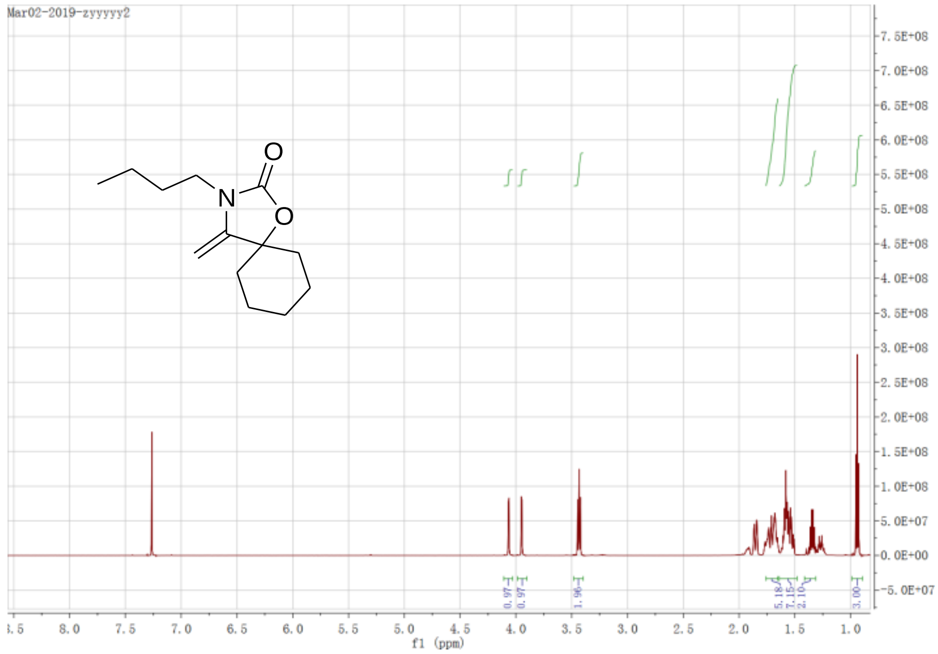
Mar02-2019-zyyyyy2
O
N
O
0.97
0.97
1.96
5.18
7.15
2.10
3.00
f1 (ppm)

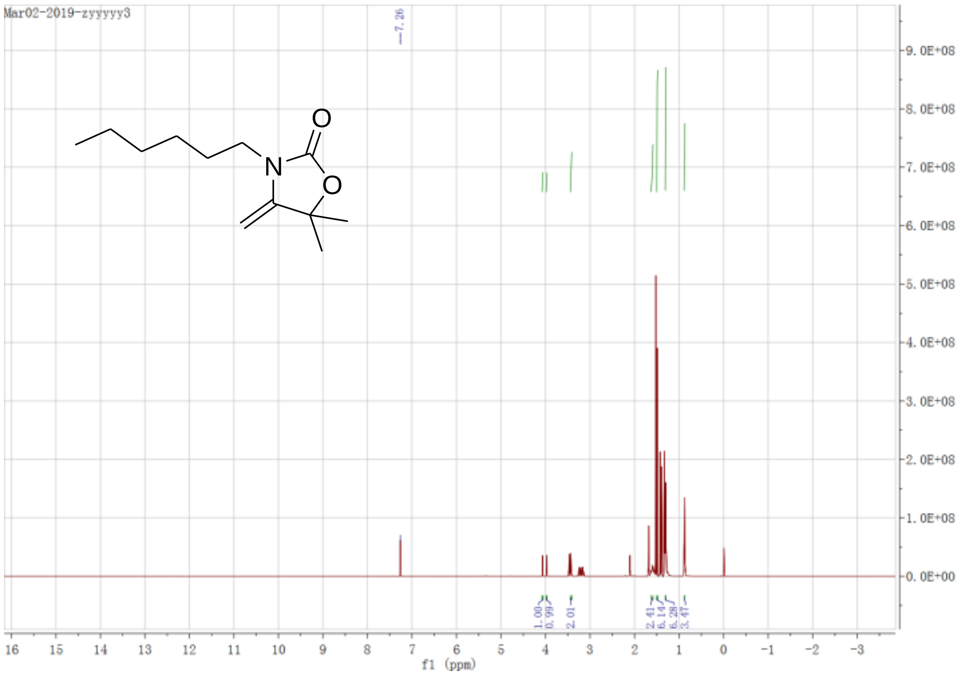
Mar02-2019-zyyyyy3
—7.26
O
N
O
1.00
0.99
2.01
2.41
6.14
6.28
3.47
f1 (ppm)

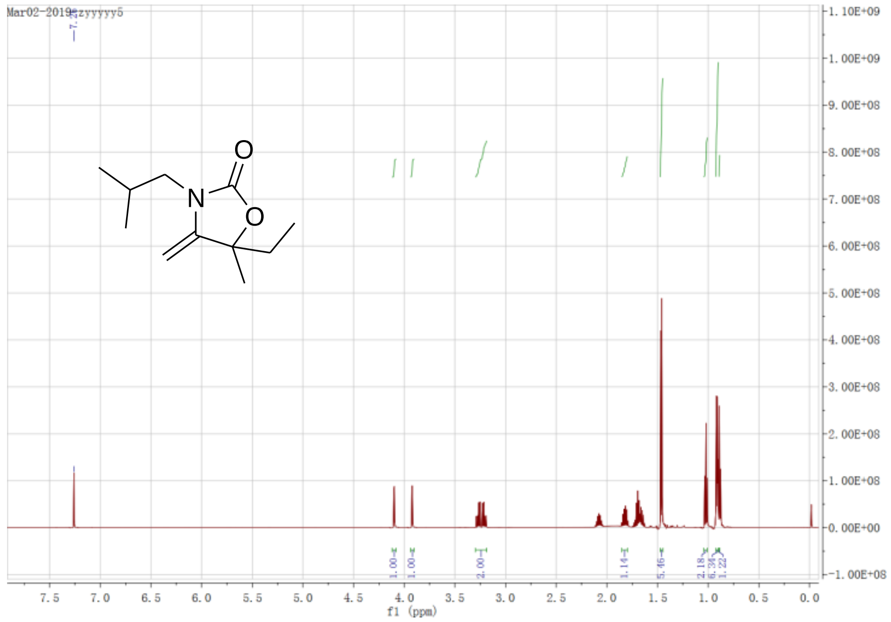
Mar02-2019-zyyyyy5
N
O
O
1.00
1.00
2.00
1.14
5.46
2.18
6.34
1.22
f1 (ppm)

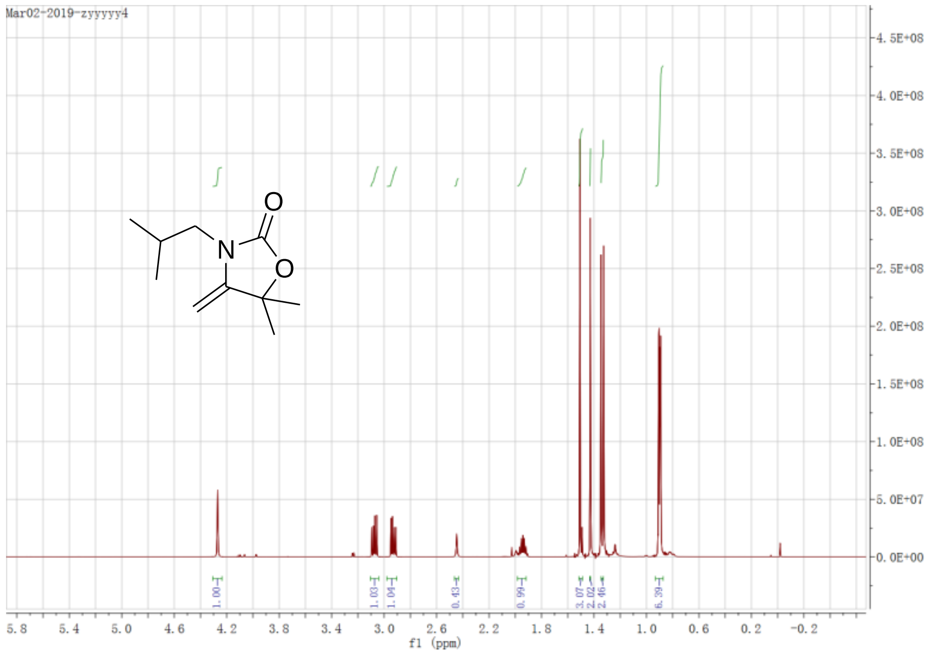
Mar02-2019-zyyyyy4
N
O
O
1.00
1.03
1.04
0.43
0.99
3.07
2.02
2.46
6.39
f1 (ppm)
